# Rescue of High Glucose Impairment of Cultured Human Osteoblasts Using Cinacalcet and Parathyroid Hormone

**DOI:** 10.1007/s00223-023-01062-7

**Published:** 2023-02-08

**Authors:** V. A. Shahen, A. Schindeler, M. S. Rybchyn, C. M. Girgis, B. Mulholland, R. S. Mason, I. Levinger, T. C. Brennan-Speranza

**Affiliations:** 1 School of Medical Sciences, Faculty of Medicine and Health, The University of Sydney, Sydney, NSW 2006 Australia; 2 Sydney Medical School, Faculty of Medicine and Health, The University of Sydney, Sydney, NSW 2006 Australia; 3 Bioengineering & Molecular Medicine Laboratory, The Children’s Hospital at Westmead and the, Westmead Institute for Medical Research, Westmead, NSW 2006 Australia; 4 School of Chemical Engineering, University of New South Wales, Sydney, NSW 2033 Australia; 5 Department of Diabetes and Endocrinology, Westmead Hospital, Sydney, Australia; 6 Department of Endocrinology, Royal North Shore Hospital, Sydney, Australia; 7 Graduate School of Medicine, Faculty of Science, Medicine and Health, University of Wollongong, Wollongong, Australia; 8 Susan Wakil School of Nursing and Midwifery, Faculty of Medicine and Health, University of Sydney, Sydney, Australia; 9 School of Life and Environmental Sciences, Faculty of Science, University of Sydney, Sydney, NSW 2006 Australia; 10 Institute for Health and Sport (IHES), Victoria University, Melbourne, VIC Australia; 11 Australian Institute for Musculoskeletal Science (AIMSS), University of Melbourne and Western Health, St Albans, VIC Australia; 12 School of Public Health, Faculty of Medicine and Health, The University of Sydney, Sydney, NSW 2006 Australia

**Keywords:** Cinacalcet, Parathyroid hormone, PTH, Diabetes, Osteoblasts

## Abstract

Patients with type 2 diabetes mellitus (T2DM) experience a higher risk of fractures despite paradoxically exhibiting normal to high bone mineral density (BMD). This has drawn into question the applicability to T2DM of conventional fracture reduction treatments that aim to retain BMD. In a primary human osteoblast culture system, high glucose levels (25 mM) impaired cell proliferation and matrix mineralization compared to physiological glucose levels (5 mM). Treatment with parathyroid hormone (PTH, 10 nM), a bone anabolic agent, and cinacalcet (CN, 1 µM), a calcimimetic able to target the Ca^2+^-sensing receptor (CaSR), were tested for their effects on proliferation and differentiation. Strikingly, CN+PTH co-treatment was shown to promote cell growth and matrix mineralization under both physiological and high glucose conditions. CN+PTH reduced apoptosis by 0.9-fold/0.4-fold as measured by Caspase-3 activity assay, increased alkaline phosphatase (ALP) expression by 1.5-fold/twofold, increased the ratio of nuclear factor κ-B ligand (RANKL) to osteoprotegerin (OPG) by 2.1-fold/1.6-fold, and increased CaSR expression by 1.7-fold/4.6-fold (physiological glucose/high glucose). Collectively, these findings indicate a potential for CN+PTH combination therapy as a method to ameliorate the negative impact of chronic high blood glucose on bone remodeling.

## Introduction

Type 2 diabetes mellitus (T2DM) is in the top ten causes of death globally [1], being associated with an increased risk of cardiovascular disease, stroke, cancer, and chronic kidney disease. The effects of T2DM on bone health are often underestimated or overlooked. Regardless of obesity or altered bone mineral density (BMD) as independent risk factors [2], bone microarchitecture and bone quality can deteriorate in patients with T2DM, leading to adverse skeletal events [3–6]. T2DM is associated with an increased fracture risk, with the relative impact reported to range from + 20% to + 300% [7]. While bone complications and fracture do not represent a primary health concern in T2DM, they add to the constellation of factors that need to be considered in terms of clinical management and healthcare costs.

The effects of T2DM on bone are often paradoxical, with the apparent bone fragility not associated with common markers of fracture risk such as BMD or the fracture risk assessment tool, FRAX, score [8–10]. Thus, while the mechanism(s) of impaired diabetic bone health are unclear, they are likely to result from the complex hyperglycemic and proinflammatory states that manifest in T2DM and can adversely influence the bone microenvironment [11]. Hyperglycemic conditions can facilitate an excess of glycosylation reactions, leading to the formation of advanced glycation end products (AGEs) [12]. This has the potential to inhibit osteoblast differentiation, suppress remodeling, and lead to a stiffening of the bone matrix [13, 14]. This can be exacerbated by oxidative stress and increased levels of proinflammatory cytokines that can accompany T2DM [15, 16].

There is no established best practice for managing the diabetic bone phenotype. Radiographs, BMD metrics, and circulating bone markers are typically only assessed following spontaneous and/or low-impact fracture(s). Even if these measures are atypical, there is limited evidence to support intervention using classical pharmacotherapies. Many anti-osteoporotic agents are bone antiresorptives, and it is unclear whether these would be beneficial in T2DM where bone turnover is already suppressed [4, 6] and BMD can be in the normal range.

A 2021 interdisciplinary expert panel highlighted the need for clinical trials to examine the efficacy and safety of available anti-osteoporotic drugs in patients with diabetes [17]. The panel also considered the use of osteoanabolic agents, such as parathyroid hormone (PTH_1–84_) and teriparatide (PTH_1–34_). These systemic interventions for osteoporosis can promote new bone formation [18, 19]; however, the treatment window for PTH is limited, with most courses lasting 18–24 months. Whether PTH and teriparatide is clinically useful in T2DM remains to be determined.

Cinacalcet (CN) is a calcimimetic drug that can act as an allosteric modulator of the calcium-sensing receptor (CaSR) [20]. Although CN is mostly used to treat patients with hypercalcemia [21, 22], it has potential to influence osteoblastic differentiation and activity. The effects on bone may be multifaceted, as CaSR modulation can also affect other bone cells such as osteoclasts [23, 24]. Thus, there are mechanistic reasons to examine the effects of CN in monoculture systems, including osteoblasts and osteoclasts [25]. CN has been reported to reduce serum intact PTH levels in hemodialysis patients with secondary hyperparathyroidism and increase BMD [26]. It has recently been used in a trial in combination with denosumab for primary hyperparathyroidism [27].

Due to the complexity of cellular interactions in the bone microenvironment, there are clear advantages to assaying the effects of bone drugs in monoculture systems. Primary human osteoblast cultures represent the gold-standard for examining the effects of pharmacotherapy on bone cells. It has been previously reported that culture under hyperglycemic conditions (high glucose) can negatively affect primary osteoblasts [28]. It was speculated that treatment with CN+PTH could lead to improved outcomes for osteoblasts grown under high glucose conditions. As such, the aim of this study was to assess the impact of CN, PTH, CN+PTH versus vehicle controls on primary human osteoblasts under physiological and high glucose conditions and examine the impact on cell growth and osteogenic differentiation.

CN has been previously shown to directly reduce parathyroid hormone stimulation in vitro, even in cells with pathologically reduced expression of the CaSR [29]. For this reason, we chose 1uM CN. A concentration of 10 nM of PTH was chosen for the current study as this is a common concentration used in in vitro studies. For example, Choudhary et al*.* has reported that 10 nM of PTH increased mineralization in vitro using marrow stromal cell (MSC) and calvarial osteoblast (COB) cultures from COX-2 knockout (KO) and wild type (WT) mice, as well as inhibiting SOST mRNA expression [30]. 10 nM PTH was also the lowest concentration of PTH found to inhibit the transcription of FGF23 in chicken bone marrow mesenchymal stem cells in vitro by Lyu et al*.*[31]*.*

## Methods

### Primary Cell Culture

**Fig. 1:**
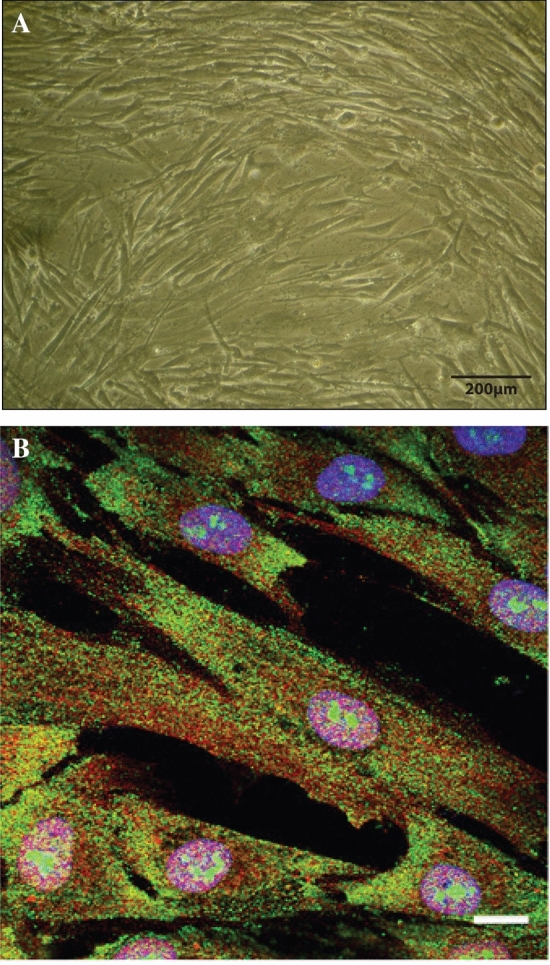
**A** Phase contrast microscopy of primary human osteoblasts (HOBs) in monolayer culture at 10X. **B** Confocal microscopy images of a monolayer culture of primary human osteoblasts taken with × 10 objective (scale bar = 10 μm) stained for CaSR (*green*) or Homer (*red*). Nuclei (*blue*) were stained with DAPI

Primary human osteoblasts (HOBs) were previously isolated from the minced trabecular ends of long bones of 18–20-week-old fetuses using a well-established model [28, 32–36], according to the National Health and Medical Research Council (NHMRC) guidelines and the project had the approval of the University of Sydney (USYD) human ethics committee (HEC) (approval number 01/02/40). These primary human osteoblasts are very well characterized with a mesenchymal appearance in monolayer culture as shown in Fig. 1A. They have high alkaline phosphatase activity, produce both osteoprotegerin (OPG) and receptor-activator of NFκB-Ligand (RANKL), and express the calcium-sensing receptor and Homer proteins [34]. When cultured on an appropriate substrate for prolonged periods, these cells form multilayers which secrete the osteocyte marker sclerostin [35]. All cultures used here are between passage 2 and 5. The effects of high glucose concentrations and osmolar controls (mannitol) on the HOBs used here have also been previously assessed [28]. Cultured primary cells were maintained in 10% fetal bovine serum (FBS) and low glucose DMEM (5 mM glucose with 3.5 g/L NaCHO_3_, 0.57 g/L Na_2_HPO_4_, and 0.006 g/L NaH_2_PO_4_, pH 7.4) (Invitrogen, Waltham, MA, USA).

Cells were treated in multi-well plates with 1 µL/mL DMSO vehicle, 1 µM CN (MedChemExpress, Monmouth Junction, NJ, USA), 10 nM PTH 1–34 (Sigma-Aldrich, St Louise, MO, USA), or 1 µM CN+10 nM PTH 1–34 in either ‘physiological glucose’ (5 mM) or ‘high glucose’ (25 mM) DMEM. The treatment period was 7 days, with media changes being performed on alternate days. Studies were performed on triplicate samples (i.e., from three separate donors) and repeated *n* = 3 or *n* = 4 times. See Fig. 1A.

### Immunofluorescence (Human Osteoblasts)

Primary human osteoblasts (HOBs) were grown on poly-l-lysine-coated coverslips for 7 days and were then fixed with 4% (w/v) paraformaldehyde followed by 100% ice-cold methanol and were processed with the following antibodies: anti-CaSR (Sigma, mouse monoclonal, clone HL1499), anti-Homer1 (Santa Cruz Biotechnology, rabbit polyclonal, clone H-174), and isotype controls. Coverslips were then washed and incubated with anti-rabbit Alexa Fluor 488 (1:750; Santa Cruz Biotechnology) and anti-mouse Cy3 (1:750; Life Technologies, Inc.) at room temperature for 60 min. To visualize the nuclei, coverslips were mounted with UltraCruz™ mounting medium containing DAPI. Slides were observed under a LSM510 Meta confocal laser microscope (Zeiss) at the Advanced Microscopy Facility (Bosch Research Institute, University of Sydney). See Fig. 1B.

### Cellular Assays

#### Live-Cell Imaging

The effects of the treatments on the rate of osteoblast proliferation were examined using the IncuCyte® Live-Cell Analysis System (Essen Bioscience, Ann Arbor, MI, USA). HOBs were seeded at a density of 0.03 × 10^6^ cells/well in 24-well plates before treatments were added. The cells were then incubated at 37° C and 4% CO_2_ in the IncuCyte Analysis instrument. Throughout the 7-day treatment period, phase scans were automatically taken at 2-h intervals using a 10 × objective. Phase object confluency was then automatically calculated and recorded by the machine and used to plot a time course for confluency. To normalize for possible differences in the initial confluence percentage between the different samples, the time required to double the initial confluence value was used for subsequent statistical analysis.

#### Cell Viability

At treatment day 7, HOBs were detached, incubated with trypan blue (Sigma-Aldrich), centrifuged, resuspended, and counted using a Neubauer Hemocytometer (Livingstone).

#### Caspase-3 Assay

At treatment day 7, a Caspase-3 assay was performed as previously described [35]. In brief, HOBs were seeded at 1 × 10^3^ in 96-well plates in physiological glucose media and allowed to attach. After 24 h, media were changed to either physiological or high glucose containing treatments as indicated. After 7 days, oxidative stress was then induced by the addition of 50 µM hydrogen peroxide and caspase activity assessed by a previously reported protocol using the caspase-3 substrate, Ac-DEVD-AFC [37]. Caspase-3 activity was used as a measure for apoptosis [38]. Cell numbers were controlled for by BCA assay (Thermo Scientific).

#### ALP Activity

At treatment day 7, HOBs in 96-well plates were assayed for alkaline phosphatase activity using a colorimetric assay as previously published [32]. Briefly, cells were lysed in 1 vol of PBS pH 7.2 containing 0.1% v/v TX-100. To the lysate, 1 vol of 1 mg/mL p-Nitrophenyl Phosphate (Sigma) in 0.2 M glycine pH 9 was added and the plate incubated at RT for 15 min. A standard curve was reacted simultaneously on the same plate using calf intestinal ALP (New England Biolabs) under the same conditions. The formation of the yellow reaction product was measured at 405 nm using a Clariostar spectrophotometer (BMG Labtech). Values were corrected for total cell protein as measured by BCA assay.

#### Alizarin Red S Staining

At treatment day 7, HOBs were washed, fixed with 4% paraformaldehyde, then stained using 2% Alizarin Red made up freshly in distilled water, pH 4.2. Cell monolayers were washed with 10% acetic acid in distilled water for 30 min at room temperature. To quantify the stain, cells were transferred into tubes, heated at 95° C, immediately placed on ice for 5 min, and centrifuged for 15 min at 20,000 g at 4° C. The absorbance of each sample was quantified using a standard curve and corrected for total cellular protein as determined by BCA assay (Thermo Fisher Scientific).

### Western Blotting

HOBs lysates from 6-well plates were separated by SDS-PAGE (Bio-Rad Laboratories, Hercules CA, USA) and were transferred to methanol-activated PVDF membranes (Thermo Fisher Scientific, Waltham, MA USA) by wet tank transfer. Membranes were blocked with BSA (Thermo Fisher Scientific) then incubated with primary antibody targeting RUNX2 (rabbit polyclonal, 1:200), OPG (mouse monoclonal, 1:200), RANKL (goat polyclonal, 1:200), OCN (mouse monoclonal, 1:200), or CaSR (mouse monoclonal, 1:2000) (all Santa Cruz Biotechnology, Dallas, TX, USA). A primary antibody targeting the housekeeping protein, β-Tubulin (mouse monoclonal, 1:200, Santa Cruz Biotech), was also used to provide means for normalization. The membranes were then incubated with the appropriate HRP-conjugated secondary antibody (Santa Cruz Biotech). Bands were detected using a ChemiDoc Imaging System (Bio-Rad) following exposure of the membrane to a chemiluminescent HRP substrate (Merck).

### Statistical Analysis

Graphing and statistical analysis were performed using GraphPad Prism software version 7.02 (GraphPad Software, California USA). A non-parametric, two-way, 2 × 4 analysis of variance (ANOVA) and post hoc Tukey’s Multiple Comparison tests were conducted compare all of the treatment groups. Graphical representations of Western blot data show the mean fold change from vehicle treatment in physiological glucose conditions ± standard error of the mean (SEM).

## Results

### CN+PTH Promotes the Growth of Cultured Human Osteoblasts

**Fig. 2:**
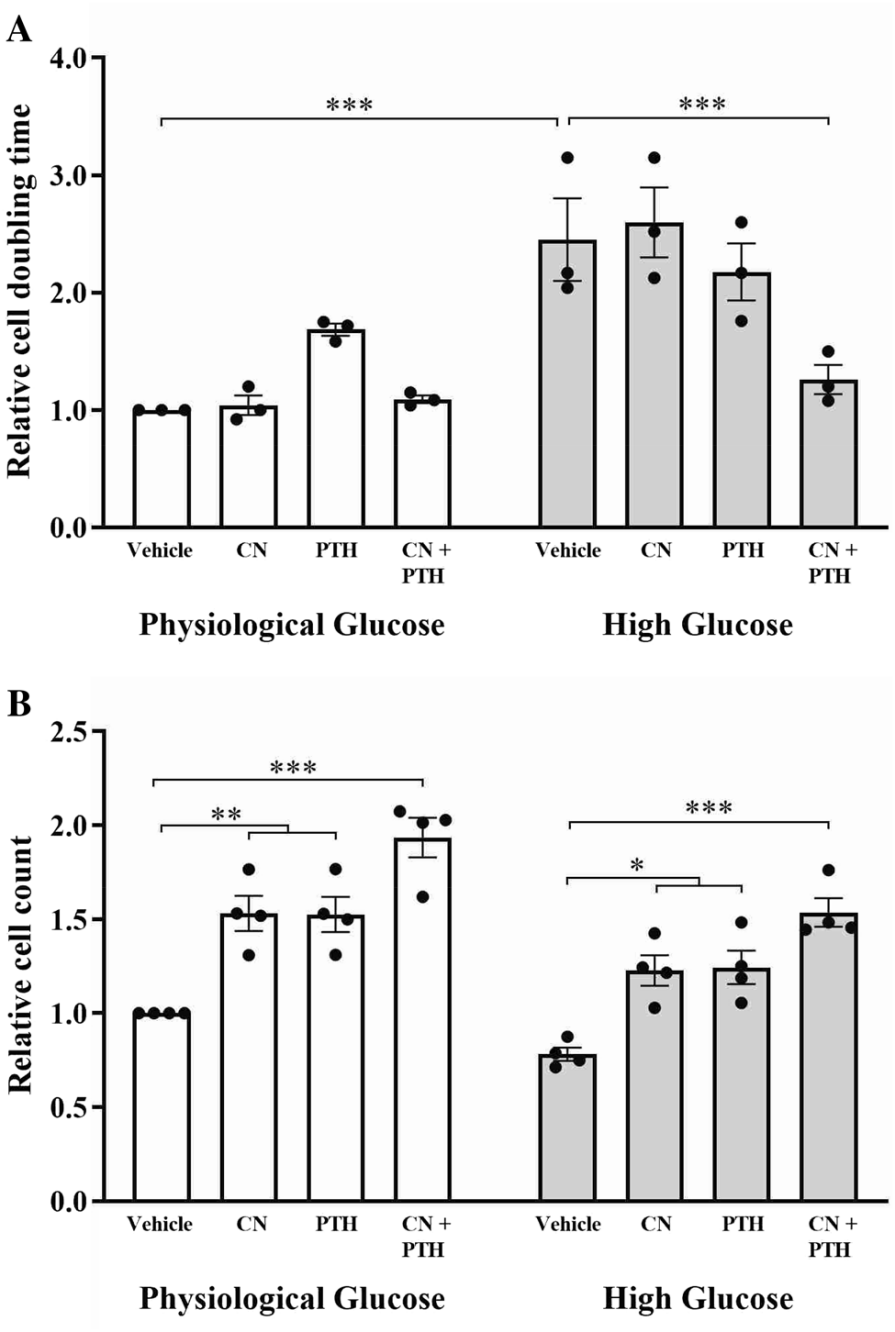
Osteoblastic proliferation and cell number. The phase object confluency (%) of primary human osteoblasts cultured in physiological (5 mM) or high (25 mM) glucose conditions and treated with a vehicle (1 µL DMSO/mL), cinacalcet (CN, 1 µM), parathyroid hormone (PTH, 10 nM), or CN+PTH for 7 days. **A** The time required for primary human osteoblasts to double their initial confluency. **B** Osteoblast number after 7 days of treatments. Data are presented as mean fold change from vehicle ± SEM. *p < 0.05, **p < 0.01, and ***p < 0.001

The effect of physiological versus high glucose was compared using the IncuCyte to determine longitudinal cell division and calculate relative doubling times. High doubling times indicated slower growth. Under high glucose conditions, this time was significantly higher for cultured human osteoblasts (Fig. 2A). While treatment with CN or PTH did not significantly rescue this slower cell growth, the combination of CN+PTH reduced the doubling times of osteoblasts grown in high glucose to that of physiological glucose (Fig. 2A). Additionally, cell numbers were counted at the conclusion of treatment at day 7 (Fig. 2B). Similar results were found, with CN+PTH increasing cell numbers (1.9-fold in physiological glucose, twofold in high glucose). CN and PTH led to increases in cell number not reflected by the IncuCyte data; however, the former assay examines growth/proliferation rates, whereas cell counts represent the product of cell proliferation at a single time point.

### The Effects of CN+PTH on Osteoblast Survival and Differentiation

**Fig. 3:**
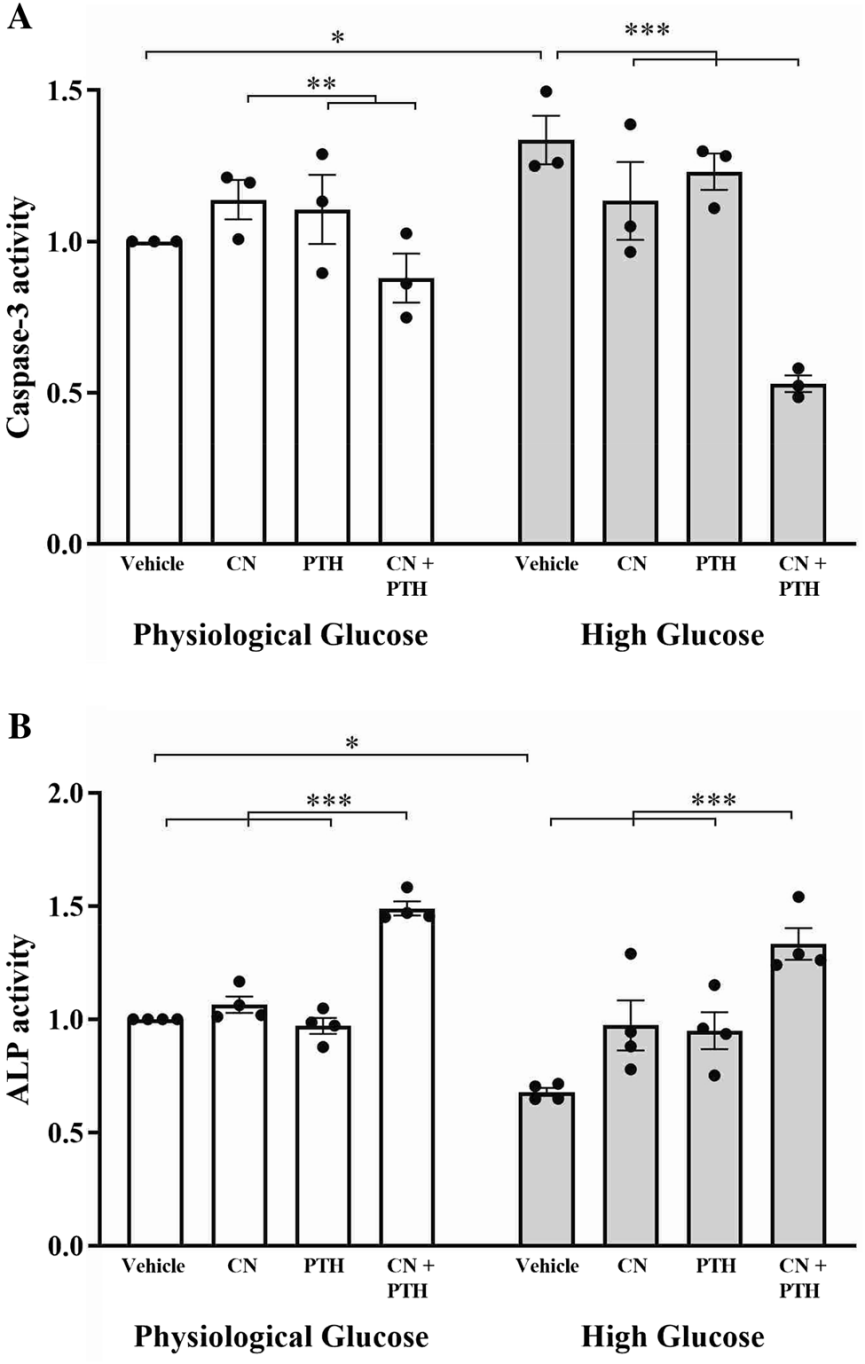
Caspase-3 and alkaline phosphatase (ALP) activity assays. Osteoblasts were treated with vehicle, CN, PTH, or CN+PTH as previously described under physiological or high glucose conditions. **A** Caspase-3 activity and **B** ALP activity in cells after 7 days of treatment. Data are presented as mean fold change from vehicle ± SEM. Note that vehicle is 5 mM (physiological) glucose. *p < 0.05 and ***p < 0.001

Biochemical assays were performed for caspase-3 and alkaline phosphatase activity. A significant 1.3-fold increase in caspase-3 activity (*p* = 0.04) was observed under high glucose conditions (Fig. 3A). This was significantly reduced by CN+PTH treatment, both compared to the elevated high glucose values as well as the basal physiological glucose levels (*p* < 0.001).

Similarly, ALP activity was significantly impaired when cells were grown under high glucose conditions (*p* = 0.02, Fig. 3B). CN+PTH increased ALP activity in both physiological (1.5-fold, *p* = 0.001) and high glucose (twofold, *p* = 0.001) environments.

### CN+PTH Treatment Rescues the Poor Mineralization Seen in High Glucose Media

**Fig. 4:**
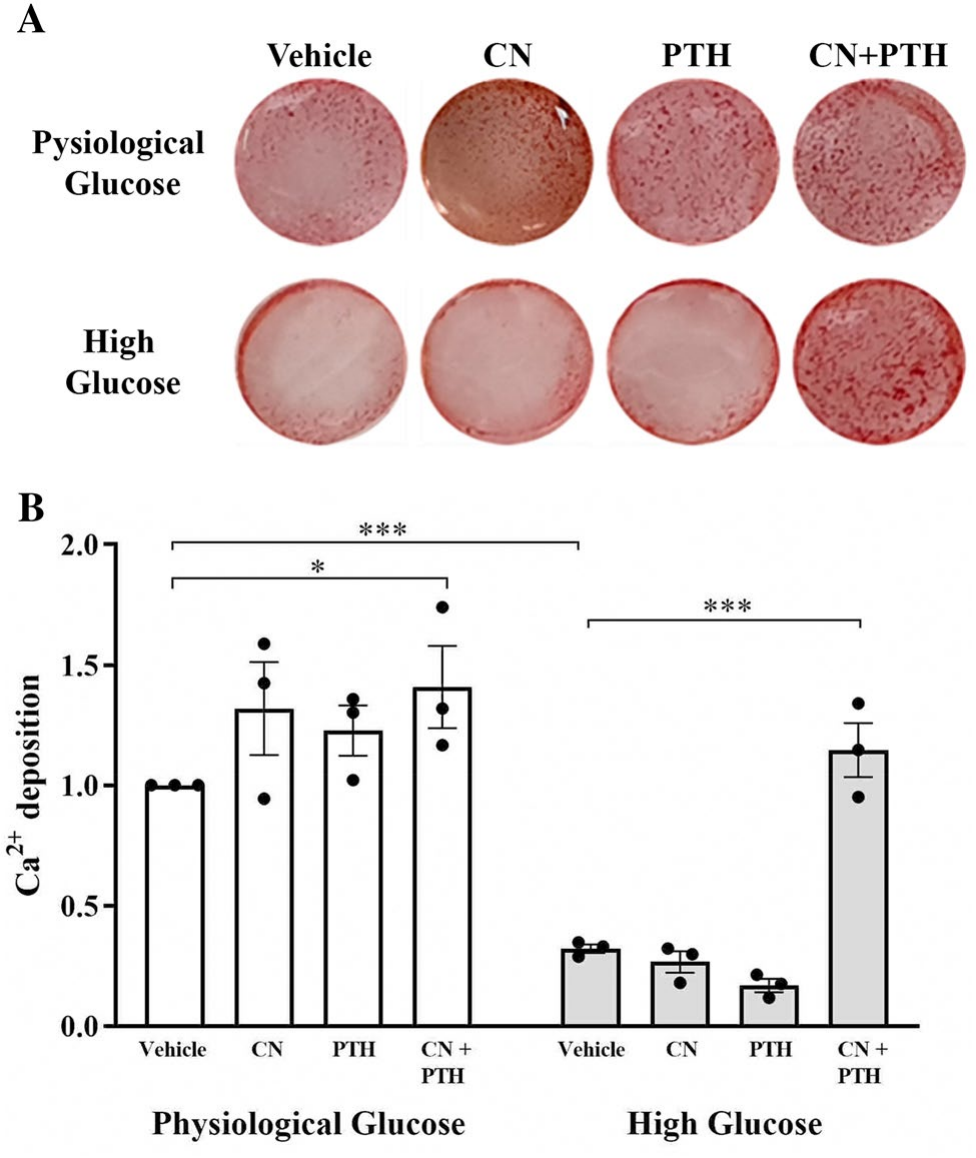
Matrix mineralization in CN, PTH, and CN+PTH-treated human osteoblasts. Osteoblasts were treated with vehicle, CN, PTH, or CN+PTH as previously described under physiological or high glucose conditions for 7 days. **A** Alizarin Red S staining at day 7 of treatment **B** Elution of stain showing quantitative Ca.^2+^ deposition levels. Data are presented as mean fold change from vehicle ± SEM. *p < 0.05 and ***p < 0.001

Cultured human osteoblasts will form a monolayer, differentiate, and express osteoblastic markers and create a mineralized matrix. This mineralized matrix is a chief feature of osteoblast maturation and thus a key functional outcome measure. In the absence of treatment, primary osteoblasts grown in standard physiological glucose media formed mineralized nodules; however, this was impaired under high glucose conditions (Fig. 4A). Staining was enhanced under both physiological glucose by CN and PTH; however, the greatest enhancement was seen with CN+PTH. This latter treatment also rescued the lack of mineralization seen in high glucose media. Quantification of staining using a dye elution method illustrated that these differences were statistically significant. For CN+PTH treatment, the increase was 1.4-fold in physiological glucose and 3.6-fold in high glucose (*p* = 0.04, Fig. 4B).

### CN+PTH Upregulates RUNX2 and OCN Expression in Human Osteoblasts

Runt-related transcription factor 2 (RUNX2) and Osteocalcin (OCN) are key markers of early- and late-stage osteogenic differentiation. Expression of these proteins was measured using Western blotting under different glucose conditions and drug treatments.

**Fig. 5:**
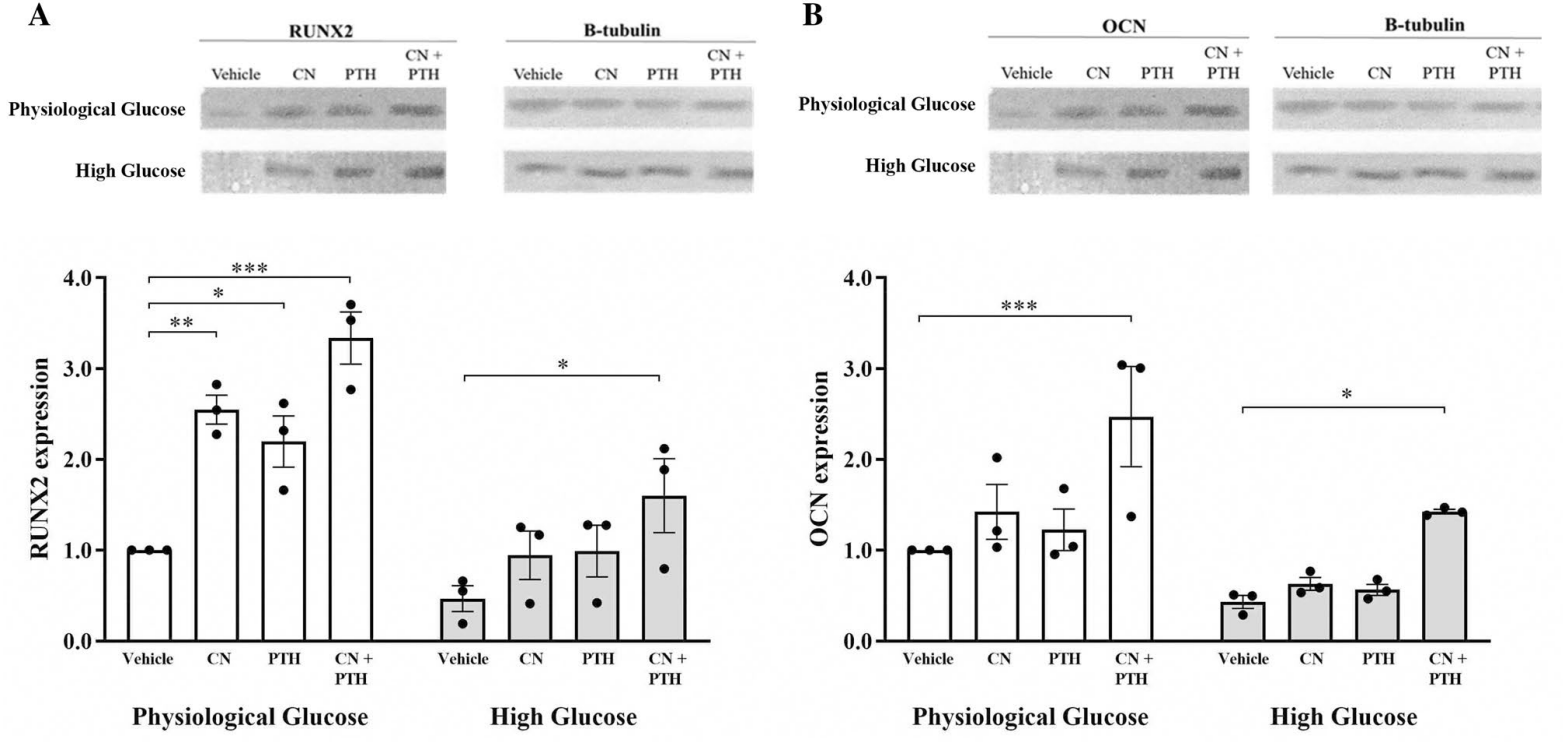
The expression levels of RUNX2 and OCN. Gene expression from immunoblots was quantified for **A** RUNX2 and **B** OCN protein expression under different glucose conditions and treatment with vehicle, CN, PTH, and CN+PTH. In panels, blots are shown above, with quantitation below as mean-fold change from physiological glucose vehicle ± SEM after normalization to the expression of the housekeeping gene, β-tubulin. *p < 0.05, **p < 0.01, ***p < 0.001

CN and PTH, used together or separately at physiological glucose levels, produced an increase in RUNX2 (Fig. 5A). CN increased RUNX2 expression by 2.5-fold (*p* < 0.01) and PTH by 2.2-fold (*p* = 0.02) when compared to the vehicle. CN+PTH increased the expression levels of RUNX2 by 3.3-fold (*p* < 0.001). When exposed to high levels of glucose, RUNX2 was reduced; however, this effect was rescued by CN + PTH (*p* < 0.001). OCN expression was similarly enhanced by CN+PTH treatment (Fig. 5B).

### CN+PTH Improves the RANKL:OPG Ratio in Human Osteoblasts

**Fig. 6:**
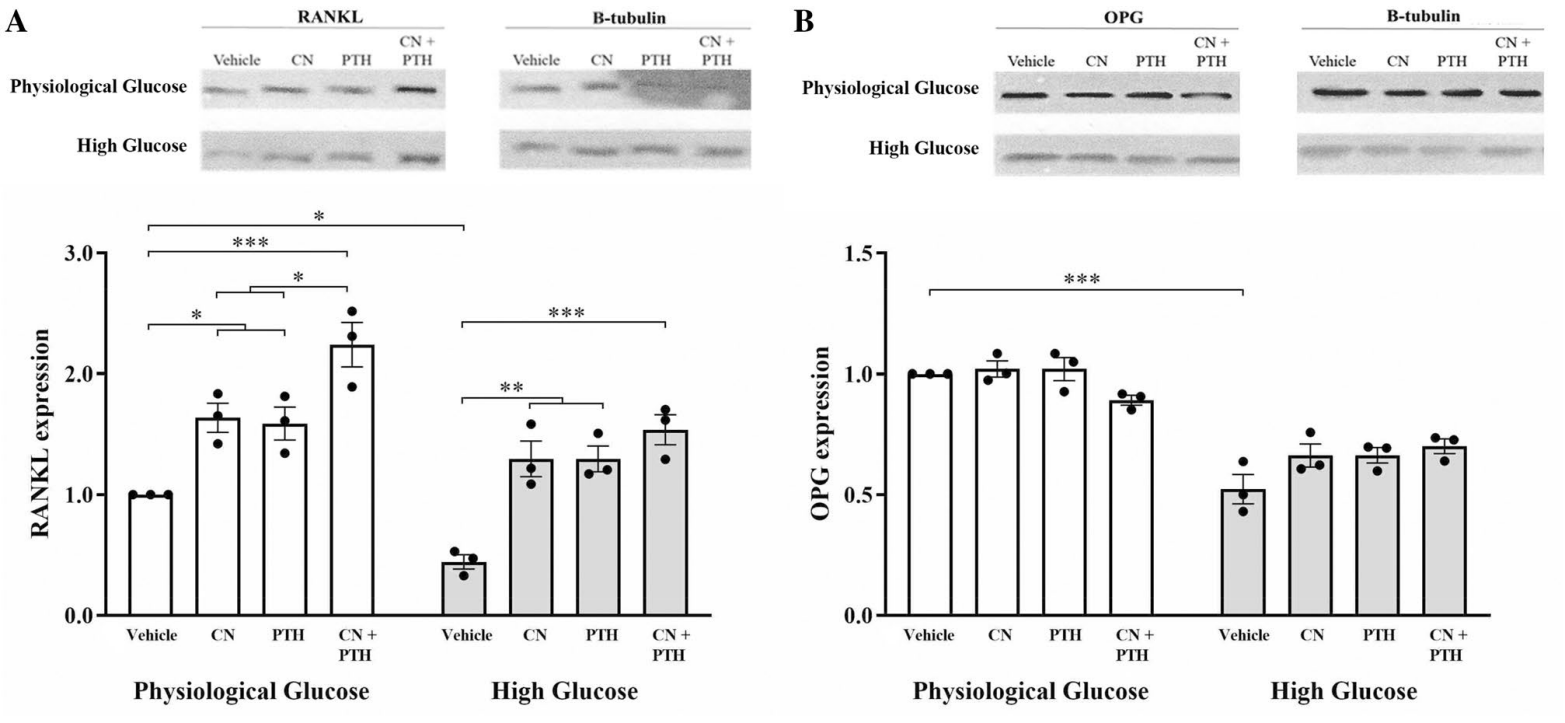
The expression levels of RANKL and OPG. Gene expression from immunoblots was quantified for **A** RANKL and **B** OPG protein expression under different glucose conditions and treatment with vehicle, CN, PTH, and CN+PTH. In panels, blots are shown above, with quantitation below as mean-fold change from vehicle ± SEM after normalization to the expression of the housekeeping gene, β-tubulin. *p < 0.05, **p < 0.01, and ***p < 0.001

Under physiological glucose conditions, CN, PTH, and CN+PTH increased RANKL expression by 1.6- to 2.2-fold, with the greatest effect with CN+PTH (Fig. 6A, B). Under high glucose conditions, RANKL expression was 0.4-fold decreased in vehicle-treated samples, but treatment with CN, PTH, and CN+PTH again increased RANKL levels by 1.9- to 2.5-fold. While OPG levels were halved under high glucose conditions, treatment with CN, PTH, and CN+PTH did not significantly change OPG (Fig. 6A, B).

Calculating RANKL:OPG ratios relative to vehicle in physiological glucose showed a 1.9-fold increase with CN, 1.4-fold increase with PTH, and 2.1-fold increase with CN+PTH (physiological glucose), and a 1.1-fold increase with vehicle, 1.8-fold increase with CN, 1.7-fold increase with PTH, and a 1.8-fold increase with CN+PTH (high glucose).

### High Glucose Levels Downregulate the CaSR, but this is Rescued Using CN+PTH

**Fig. 7:**
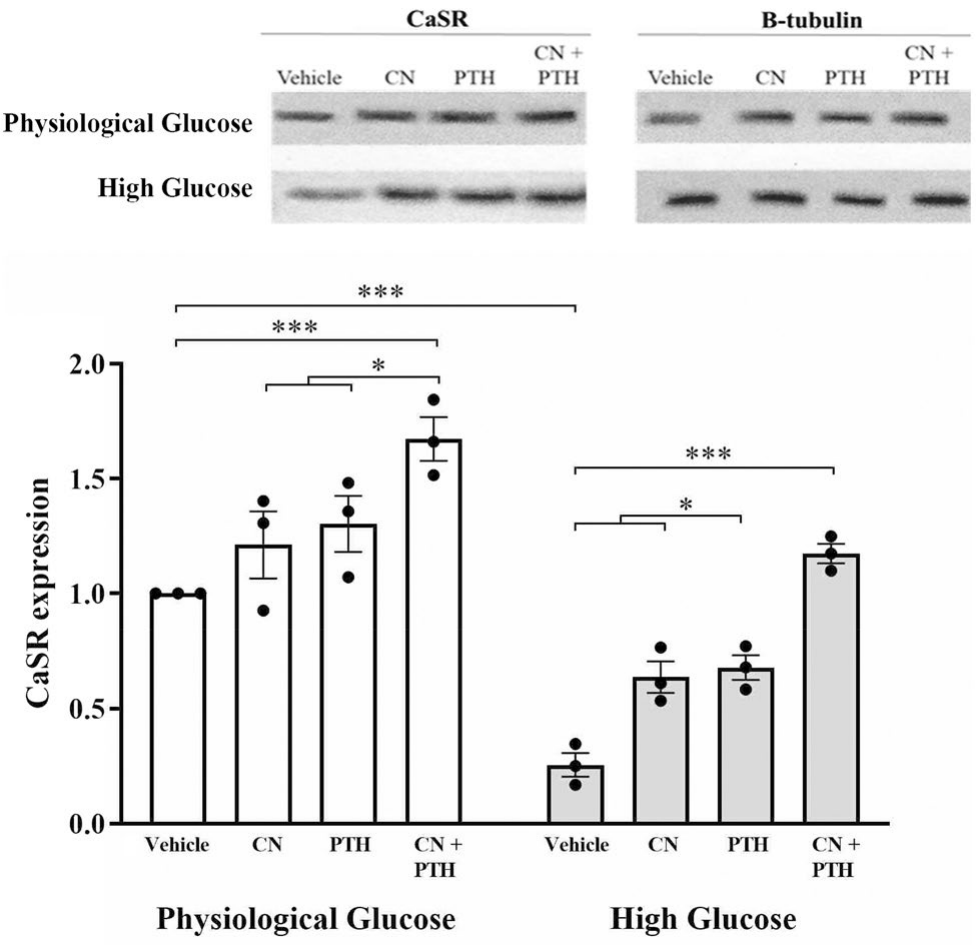
The expression levels of CaSR. Gene expression from immunoblots was quantified for CaSR under different glucose conditions and treatment with vehicle, CN, PTH, and CN+PTH. In panels, blots are shown above, with quantitation below as mean-fold change from vehicle ± SEM after normalization to the expression of the housekeeping gene, β-tubulin. ***p < 0.001

In high glucose media, CN and PTH were found to significantly elevate CaSR expression by 1.5-fold and 1.7-fold, respectively (Fig. 7). CN+PTH showed a greater effect on CaSR expression that was significantly elevated vs no treatment vehicle controls under physiological (1.7-fold increase) and high glucose (3.6-fold increase) conditions (Fig. 7).

## Discussion

**Fig. 8:**
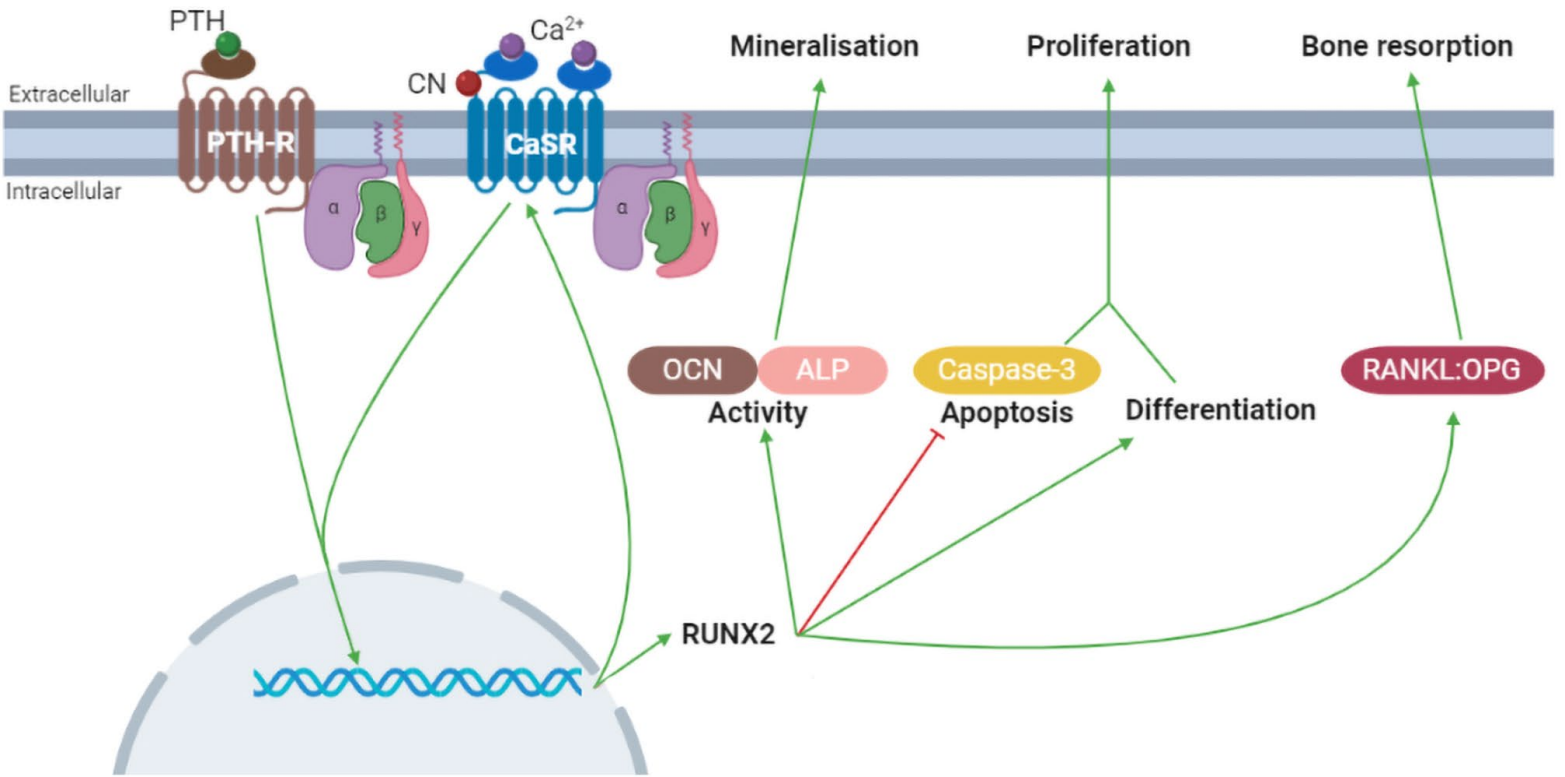
The effects of cinacalcet (CN) and parathyroid hormone (PTH) on osteoblasts. CN acts on and increases the sensitivity of the Ca^2+^ sensing receptor (CaSR) to Ca^2+^. Ca^2+^ activates the CaSR and PTH activates the PTH receptor (PTH-R). The activation of the receptors leads to several transcriptional effects. Firstly, receptor activation upregulates runt-related transcription factor 2 (RUNX2). This increases the expression of osteocalcin (OCN) and enhances alkaline phosphatase (ALP) activity, which are hallmarks of osteoblastic activity. The increase in activity then results in enhanced mineralization and hence bone formation. RUNX2 also stimulates differentiation and maturation and inhibits apoptosis by reducing caspase-3 activity. This stimulates osteoblastic proliferation, leading to more cells. Secondly, receptor activation upregulates receptor activator of nuclear factor κ-B ligand (RANKL). This increases the ratio of RANKL to its inhibitor, osteoprotegerin (OPG), which can then stimulate osteoclastic activity and bone resorption. Finally, activating the CaSR and PTH-R also leads to the upregulation of the CaSR, creating a positive feedback loop

This study provides a comprehensive analysis of the impact of intervention with PTH, CN, and PTH+CN on primary osteoblasts cultured under physiological and high glucose conditions. We have previously shown that even 2 h of varying glucose concentrations affects these HOBs with higher concentrations (up to 20 mM) reducing ALP activity, cell viability, and osteocalcin RNA expression, and increasing apoptosis (via the same Caspase assay we have used in the current study) [28]. The treatment period chosen for the current study was 7 days to allow the high glucose environment to elicit an impact that is more likely to mimic that of chronic hyperglycemia in T2DM within the time constraints of an in vitro cell culture model. Our findings support the concept of hyperglycemia negatively impacting on osteoblasts. Drug treatments promoted a range of benefits, including increased proliferation, differentiation, and function (mineralization). In many cases, the improvements produced by PTH+CN were greater under the problematic high glucose conditions. The pathways affected by pharmaceutical intervention are shown schematically in Fig. 8. One of the most interesting findings to come from the current study was the upregulation of the CaSR in HOBs exposed to either PTH alone, CN alone, and even more markedly with the PTH+CN combination treatment. The body requires that circulating Ca^2+^ concentrations to be kept within a very narrow range, requiring that any cells that need to detect changes in this near-consistent parameter have a remarkable sensitivity to fluctuations in extracellular Ca^2+^ concentrations. It is thus very likely that calciotropic hormones, such as PTH, and pharmaceutical agents that have been developed to work with Ca^2+^ homeostasis, indeed upregulate the expression of the very receptor that detects these changes—the CaSR. Another calcimimetic, NPS R-568 has been shown to upregulate both mRNA and protein levels of the CaSR in the parathyroid gland in rats [39]. This co-operation underpins the sophistication of the Ca^2+^ homeostatic system.

PTH+CN may also indirectly improve bone turnover via improving the RANKL:OPG ratio, as bone turnover can be impaired in T2DM [28]. While the impacts of CN and PTH were not redundant, for most outcomes, the combination produced an additive rather than overtly synergistic effect.

The mechanisms by which high glucose leads to poor bone health is not well understood [40]. Published studies have used simulated models of diabetes (in vitro and in vivo) to examine how hyperglycemia affects the behavior of bone cells [41, 42]. The effects of high glucose on osteoblasts do not appear to be related to osmolarity, as prior studies found no impact with a comparable concentration of D-mannitol [28]. Most prior studies have employed immortalized osteoblasts or primary rodent cells, rather than primary human osteoblasts. While cell lines are a convenient and accessible research tool, they should not be considered appropriate replacements for primary osteoblasts [43]. Our findings of the effects of high glucose are consistent with those of Garcia-Hernandez et al., who treated primary human osteoblasts with up to 24 mM glucose and reported impaired matrix mineralization, osteogenic protein markers, and RANKL:OPG ratio [44].

Future directions for this research will focus on mechanism and clinical translation. The effects of high glucose on osteoblasts are potentially mediated by elevated cytokine expression [44]. However, the correlation and relevance of in vitro cytokine expression and in vivo physiology are unclear. Other factors may also play a role such as the glycoprotein Fetuin-A, which can be upregulated by cinacalcet [45], and are associated with impaired insulin sensitivity and glucose tolerance. While there is utility in candidate approaches, unbiased approaches such as transcriptome sequencing may uncover novel mechanisms and druggable targets. Our data support further investigation into the clinical use of PTH/teriparatide and cinacalcet in patients with diabetes and osteoporosis. Further data supporting prospective studies could be potentially gained from subgroup analysis of prior trials. For example, reduced fractures were seen with cinacalcet treatment in end-stage renal disease patients [46]; presumably, a proportion of these patients were diabetic as it is one of the most common causes of renal impairment.

## References

[1] Lin X (2020). Global, regional, and national burden and trend of diabetes in 195 countries and territories: an analysis from 1990 to 2025. Sci Rep.

[2] Fruhbeck G (2013). Obesity: the gateway to ill health - an EASO position statement on a rising public health, clinical and scientific challenge in Europe. Obes Facts.

[3] Warming L, Hassager C, Christiansen C (2002). Changes in bone mineral density with age in men and women: a longitudinal study. Osteoporos Int.

[4] Reyes-Garcia R (2013). Serum levels of bone resorption markers are decreased in patients with type 2 diabetes. Acta Diabetol.

[5] Lee BK (2016). Comparison of age of onset and frequency of diabetic complications in the very elderly patients with type 2 diabetes. Endocrinol Metab (Seoul).

[6] Purnamasari D (2017). Low bone turnover in premenopausal women with type 2 diabetes mellitus as an early process of diabetes-associated bone alterations: a cross-sectional study. BMC Endocr Disord.

[7] Dede AD (2014). Type 2 diabetes mellitus and fracture risk. Metabolism.

[8] Tuominen JT (1999). Bone mineral density in patients with type 1 and type 2 diabetes. Diabetes Care.

[9] Schwartz AV (2011). Association of BMD and FRAX score with risk of fracture in older adults with type 2 diabetes. JAMA.

[10] Giangregorio LM (2012). FRAX underestimates fracture risk in patients with diabetes. J Bone Miner Res.

[11] Shahen VA (2020). Multifactorial effects of hyperglycaemia, hyperinsulinemia and inflammation on bone remodelling in type 2 diabetes mellitus. Cytokine Growth Factor Rev.

[12] Malik P (2015). Role of receptor for advanced glycation end products in the complication and progression of various types of cancers. Biochim Biophys Acta.

[13] McCarthy AD (2004). Advanced glycation endproducts interefere with integrin-mediated osteoblastic attachment to a type-I collagen matrix. Int J Biochem Cell Biol.

[14] Snedeker JG, Gautieri A (2014). The role of collagen crosslinks in ageing and diabetes - the good, the bad, and the ugly. Muscles Ligaments Tendons J.

[15] Bai XC (2004). Oxidative stress inhibits osteoblastic differentiation of bone cells by ERK and NF-kappaB. Biochem Biophys Res Commun.

[16] Lam J (2000). TNF-alpha induces osteoclastogenesis by direct stimulation of macrophages exposed to permissive levels of RANK ligand. J Clin Invest.

[17] Chiodini I (2021). Management of bone fragility in type 2 diabetes: perspective from an interdisciplinary expert panel. Nutr Metab Cardiovasc Dis.

[18] Hodsman AB (2003). Efficacy and safety of human parathyroid hormone-(1–84) in increasing bone mineral density in postmenopausal osteoporosis. J Clin Endocrinol Metab.

[19] Aslan D (2012). Mechanisms for the bone anabolic effect of parathyroid hormone treatment in humans. Scand J Clin Lab Invest.

[20] Hebert SC (2006). Therapeutic use of calcimimetics. Annu Rev Med.

[21] Block GA (2004). Cinacalcet for secondary hyperparathyroidism in patients receiving hemodialysis. N Engl J Med.

[22] Torres PU (2006). Cinacalcet HCl: a novel treatment for secondary hyperparathyroidism caused by chronic kidney disease. J Ren Nutr.

[23] Dvorak MM (2007). Constitutive activity of the osteoblast Ca2+-sensing receptor promotes loss of cancellous bone. Endocrinology.

[24] Dvorak MM (2004). Physiological changes in extracellular calcium concentration directly control osteoblast function in the absence of calciotropic hormones. Proc Natl Acad Sci USA.

[25] Shalhoub V (2003). In vitro studies with the calcimimetic, cinacalcet HCl, on normal human adult osteoblastic and osteoclastic cells. Crit Rev Eukaryot Gene Expr.

[26] Tsuruta Y (2013). Effects of cinacalcet on bone mineral density and bone markers in hemodialysis patients with secondary hyperparathyroidism. Clin Exp Nephrol.

[27] Leere JS (2020). Denosumab and cinacalcet for primary hyperparathyroidism (DENOCINA): a randomised, double-blind, placebo-controlled, phase 3 trial. Lancet Diabetes Endocrinol.

[28] Levinger I (2016). Glucose-loading reduces bone remodeling in women and osteoblast function in vitro. Physiol Rep.

[29] Kawata T (2006). Direct in vitro evidence of the suppressive effect of cinacalcet HCl on parathyroid hormone secretion in human parathyroid cells with pathologically reduced calcium-sensing receptor levels. J Bone Miner Metab.

[30] Choudhary S (2008). Anabolic effects of PTH in cyclooxygenase-2 knockout osteoblasts in vitro. Biochem Biophys Res Commun.

[31] Lyu Z (2022). Fibroblast growth factor 23 inhibits osteogenic differentiation and mineralization of chicken bone marrow mesenchymal stem cells. Poult Sci.

[32] Brennan TC, Rybchyn MS, Green W, Atwa S, Conigrave AD, Mason RS (2009). Osteoblasts play key roles in the mechanisms of action of strontium ranelate. Br J Pharmacol.

[33] Rybchyn MS, Green WL, Conigrave AD, Mason RS (2009). Involvement of both GPRC6A and the calcium-sensing receptor in strontium ranelate-induced osteoclastogenic signal expression and replication in primary human osteoblasts. Bone.

[34] Rybchyn MS (2019). Homer1 mediates CaSR-dependent activation of mTOR complex 2 and initiates a novel pathway for AKT-dependent beta-catenin stabilization in osteoblasts. J Biol Chem.

[35] Rybchyn MS (2011). An Akt-dependent Increase in canonical Wnt signaling and a decrease in sclerostin protein levels are involved in strontium ranelate-induced osteogenic effects in human osteoblasts. J Biol Chem.

[36] Slater M (1994). Modulation of growth factor incorporation into ECM of human osteoblast-like cells in vitro by 17 beta-estradiol. Am J Physiol.

[37] Grebenová D (2003). Mitochondrial and endoplasmic reticulum stress-induced apoptotic pathways are activated by 5-aminolevulinic acid-based photodynamic therapy in HL60 leukemia cells. J Photochem Photobiol B.

[38] McIlwain DR, Berger T, Mak TW (2015). Caspase functions in cell death and disease. Cold Spring Harb Perspect Biol.

[39] Mizobuchi M (2004). Calcimimetic compound upregulates decreased calcium-sensing receptor expression level in parathyroid glands of rats with chronic renal insufficiency. J Am Soc Nephrol.

[40] Rizzoli R, Biver E, Brennan-Speranza TC (2021). Nutritional intake and bone health. Lancet Diabetes Endocrinol.

[41] Li Y (2020). Impact of diabetes mellitus simulations on bone cell behavior through in vitro models. J Bone Miner Metab.

[42] Aswamenakul K (2020). Proteomic study of in vitro osteogenic differentiation of mesenchymal stem cells in high glucose condition. Mol Biol Rep.

[43] Czekanska EM (2014). A phenotypic comparison of osteoblast cell lines versus human primary osteoblasts for biomaterials testing. J Biomed Mater Res A.

[44] Garcia-Hernandez A (2012). High glucose concentrations alter the biomineralization process in human osteoblastic cells. Bone.

[45] Messa P (2007). Calcimimetic increases osteoprotegerin and decreases fetuin-A levels in dialysis patients. Nephrol Dial Transplant.

[46] Investigators ET (2012). Effect of cinacalcet on cardiovascular disease in patients undergoing dialysis. N Engl J Med.

